# Giving a Body a Different Face—How Men and Women Evaluate Their Own Body vs. That of Others

**DOI:** 10.3389/fpsyg.2022.853398

**Published:** 2022-05-02

**Authors:** Mona M. Voges, Hannah L. Quittkat, Benjamin Schöne, Silja Vocks

**Affiliations:** ^1^Department of Clinical Psychology and Psychotherapy, Institute of Psychology, Osnabrück University, Osnabrück, Germany; ^2^Department of Experimental Psychology, Institute of Psychology, Osnabrück University, Osnabrück, Germany

**Keywords:** body evaluation, gender difference, identity, body image, self-deprecating bias, double standards

## Abstract

Eating disorders affect women more than men. Women reportedly dislike their body shape more and appreciate it less than do men. One factor influencing body image might be the application of different standards for oneself than for other people when evaluating bodies. To investigate this possibility, we determined whether the application of double standards is different between men and women. We presented 57 women and 54 men (aged 18–30 and of average weight) with pictures of their own bodies and pictures of average weight, overweight, and “ideal” bodies attached to the participants’ own face and to another person’s face. Participants were instructed to evaluate their emotional reaction to the pictures and then rate the various pictures on aspects of attractiveness, body fat, and muscle mass. The degree of the double standard was defined as the difference between ratings of what appeared to be one’s own body and what appeared to be someone else’s according to the presented face. The analyses revealed, firstly, that both genders applied self-deprecating double standards when viewing overweight and average-weight bodies. Women, but not men, also showed self-deprecating double standards when viewing the ideal body and their own body. By contrast, men applied fewer double standards when viewing the ideal body and self-enhancing double standards when viewing their own body. The study suggests that young, average-weight men are more or less satisfied with their own bodies, whereas young, average-weight women tend to apply a stricter standard for themselves than for others, thus devaluing their own bodies. This vulnerability to body image is hypothesized as contributing to the prevalence of eating disorders in women.

## Introduction

Body dissatisfaction is not only a prominent risk factor for the development and maintenance of eating disorders (Glashouwer et al., 2019), but also seems to be a risk factor for or a contributing factor to low self-esteem and depression (Brechan and Kvalem, 2015), social anxiety, post-traumatic stress disorder (Dyer et al., 2015), borderline personality disorder (Dyer et al., 2013), and body dysmorphic disorder (Phillipou et al., 2019). Therefore, detecting factors that contribute to and influence body dissatisfaction could be useful to inform the development of preventive and therapeutic strategies for body dissatisfaction and its associated outcomes.

One factor associated with body dissatisfaction is gender. Research indicates that most women in Western societies have internalized a hard-to-achieve thin body as ideal and show some degree of body dissatisfaction (Coker and Abraham, 2014; Behrens et al., 2021), which is described as normative discontent in women (Rodin et al., 1984). Body dissatisfaction is typically lower in men than in women (Quittkat et al., 2019). Furthermore, body appreciation, which is a manifestation of a positive body image including favorable opinions about, acceptance of, and respect for one’s own body, along with protective thought patterns that reject unrealistic body appearance ideals (Tylka and Wood-Barcalow, 2015), is usually higher in men than in women (He et al., 2020b). Given that a slim body ideal exists for both genders, a higher body mass index (BMI) is associated with higher body dissatisfaction (Calzo et al., 2012) and lower body appreciation (He et al., 2020a). The association between BMI and body dissatisfaction can already be observed in adolescent boys and girls (Calzo et al., 2012). However, for girls, but not for boys, even a healthy weight is associated with greater weight and shape concerns (Calzo et al., 2012), and the link between body appreciation and BMI is weaker for men than for women (He et al., 2020a).

Different factors may contribute to such gender differences in body image. One factor might be that there is one clear thin ideal for female bodies, with which women are often confronted in the media, while the media presentation of male bodies is more diverse and less pervasive (Buote et al., 2011). A further factor may be that beauty and body size are more relevant for self-worth in women than in men, meaning that women invest more in body appearance (Owens et al., 2010). Stereotypes emphasizing body functionality in men and body appearance in women might foster higher body dissatisfaction and lower body appreciation in women than in men (Murnen and Smolak, 2019). However, a high drive for muscularity, which is also linked to unhealthy exercise and dietary behaviors, is more likely to occur in men than in women, as it is seen as a sign of masculinity (Murnen and Smolak, 2019). Furthermore, in recent years, social media has become increasingly important for young people, and might convey messages about dysfunctional body attitudes that promote body image disorders in vulnerable women and men (Lonergan et al., 2021). In sum, while society and media might contribute to gender differences in body dissatisfaction, the specific mechanisms maintaining different body attitudes in women and men are unclear.

According to the cognitive-behavioral theories of eating disorders (Williamson et al., 2004) and body dysmorphic disorder (Neziroglu et al., 2008), body dissatisfaction might be fostered by cognitive biases in attentional, memory, perceptual, and evaluative processes that lead to increased negative emotions toward one’s own body. In line with these theories, previous study findings support the existence of different cognitive biases (Rodgers and DuBois, 2016) and increased negative emotions toward one’s own body in persons with high body image concerns (Griffen et al., 2018). With regard to evaluative processes, appearance-related interpretation biases have been found, meaning that ambiguous scenarios are more likely to be interpreted as body-related or as negative (Dietel et al., 2020). According to cognitive-behavioral theory, such biases might result from the activation of body-related schemas, which lead to a more schema-congruent processing of the situation (Williamson et al., 2004). In line with this thesis, it has been reported that individuals with eating disorders tend to overestimate their body size (Mölbert et al., 2017), which likely results from dysfunctional attitudes that distort the person’s own evaluation rather than due to visual deficits (Mölbert et al., 2018). Also in line with a schema-congruent processing, women with body image concerns rate their own bodies as less attractive than how other persons rate their own bodies (Horndasch et al., 2015). However, cognitive biases are not necessarily dysfunctional. These biases may also be self-enhancing, as demonstrated by attractiveness ratings in young women with low levels of body concerns (Jansen et al., 2006) who rated their own bodies as more attractive than other bodies and as more attractive than how other persons rated them. In contrast, young women with high levels of body image concerns showed no cognitive bias as they rated their own bodies to be equally attractive as other bodies in the same way as other persons rated them (Jansen et al., 2006; Alleva et al., 2013).

Most of the research on evaluative biases and body image has been conducted in women, rendering it difficult to draw conclusions about such biases in men. A study comparing body evaluations of men and women found that both women and men rated their own bodies as more attractive than how other persons rated them, suggesting a self-enhancing bias in attractiveness ratings in both genders (Donaghue and Smith, 2008). In contrast, Buote et al. (2011) found a gender difference in cognitive style regarding body image in young women and men. The authors reported that even though women, but not men, knew that the thin ideal is generally hard to reach, they believed that it was personally more attainable for them, suggesting the application of a double standard. Double standards refer to the use of different standards to judge a characteristic of two or more persons merely because of a differentiating feature (Foschi, 1996), such as gender or identity. According to Buote et al. (2011), women might apply stricter standards for themselves than for others in the opportunity to influence one’s own body shape. Thus, the use of different standards for oneself than for others in body evaluation might constitute one specific evaluative bias that contributes to gender differences in body dissatisfaction.

To examine whether women apply different standards for oneself than for others in terms of body evaluation and how their standards differ from men, Voges et al. (2019b) presented different bodies, once with the participant’s own face and once with another face, to women and men who were asked to rate the bodies depending on identity. According to the cognitive-behavioral theory of eating disorders (Williamson et al., 2004), one’s own face, as an identity cue, might activate different body schemas compared to another person’s face, leading to a different point of view and different evaluations of the bodies. Using this study design, it was shown that women mostly apply the same standard to bodies (no difference in rating because of identity), with the exception of an overweight body which was rated more negatively when it was presented with one’s own face (Voges et al., 2019a). Thus, women only showed stricter standards for oneself than for others in the case of an overweight body, and not generally. Similarly, men showed self-deprecating double standards for overweight bodies but also self-enhancing double standards in the case of an ideal athletic body, rating it as more attractive with one’s own face (Voges et al., 2019b). For women, a more self-deprecating evaluation of average-weight and overweight bodies was associated with a higher degree of body dissatisfaction, but no further associations were found. However, the idea that body dissatisfaction is linked to more self-deprecating double standards is underpinned by findings in women with anorexia nervosa and bulimia nervosa who consistently rated bodies with one’s own face as fatter than the same bodies with another face (Voges et al., 2018). A limitation of these studies lies in the use of artificially produced cartoon-like bodies that were digitally created, thus limiting the ecological validity and in turn potentially hampering the identification with these bodies. Thus, the use of real body stimuli might result in more pronounced double standards for both genders. Furthermore, the aforementioned studies did not examine participants’ evaluation of their own real body with one’s own or another identity, thus precluding the possibility to draw conclusions about double standards for one’s own body. Such double standards regarding one’s own body might be more strongly associated with body image disturbances than double standards regarding other bodies.

Therefore, the aim of the present study was to overcome these limitations and analyze the application of double standards for real body stimuli, including one’s own body. For this purpose, frontal photographs of the participants’ bodies and of other average-weight, overweight, and “ideal” female and male bodies were taken. Female and male participants were presented with the same-sex bodies, once with the participant’s face and once with another face. Participants had to indicate their emotional reactions to the bodies in terms of valence and arousal, and to evaluate the bodies with regard to attractiveness, body fat, and muscle mass. Attractiveness and body fat were chosen because previous studies found biases in the ratings of these variables that might be linked to body dissatisfaction (Jansen et al., 2006; Alleva et al., 2013; Mölbert et al., 2017). Muscle mass was added because the male body image depends not only on body size but also on muscularity (Murnen and Smolak, 2019). However, muscularity is evidently becoming increasingly important for women too, as reflected by recent social media trends such as “fitspiration” (Tiggemann and Zaccardo, 2015), which propose a slender but muscular body as an ideal for women (Hartmann et al., 2018). Emotional ratings were included to examine whether double standards in body evaluation are also accompanied by a different emotional activation, which is suggested by the cognitive-behavioral theory of eating disorders (Williamson et al., 2004).

The degree of the double standard was defined as the difference between ratings of what appeared to be one’s own and someone else’s body according to the presented face. We hypothesized that both women and men would show self-deprecating double standards in all dependent variables in the case of an overweight body, as was found for both genders in a previous study employing cartoon-like bodies as stimuli (Voges et al., 2019b). Thus, women and men should evaluate the overweight body as less attractive, with more body fat, and with less muscle mass, and should evaluate a higher negative emotional reaction, i.e., higher arousal and lower valence, when the body is presented with one’s own face compared to with the other face. We expected that women would not show any further double standards, but would apply the same standard to the other images (Voges et al., 2019a). In contrast, we expected that men should show some self-enhancing double standards in the case of the ideal male body, as was found in a previous study using cartoon-like bodies (Voges et al., 2019b), and possibly also in the case of one’s own body as a manifestation of their positive attitude toward one’s own body (He et al., 2020b). Thus, men should rate the ideal body and possibly one’s own body as more attractive and with more muscle mass, and evaluate a higher positive emotional reaction, i.e., higher arousal and higher valence, when the body is presented with one’s own face compared to with the other face. Furthermore, we hypothesized that body dissatisfaction would be associated with more self-deprecating double standards, especially in women, in view of previous findings that women with eating disorders show more pronounced self-deprecating double standards (Voges et al., 2018). In contrast, in both genders, body appreciation should be associated with more self-enhancing double standards and fewer self-deprecating double standards.

## Materials and Methods

### Participants

Participants were recruited through mailing lists, university lectures, press releases, Facebook advertisements, and flyers. Inclusion criteria were as follows: aged between 18 and 30 years, a BMI of 18.5–30 kg/m^2^, and the absence of a mental disorder based on self-report with two yes/no questions (“Do you currently suffer from a diagnosed mental disorder?” and “Are you currently in treatment because of a diagnosed mental disorder?”). The BMI criterion was chosen based on World Health Organization (WHO) criteria for BMI and statistical distributions of BMI in women and men aged 18–30 years in Western societies. According to the WHO, normal weight for both genders is defined as a BMI of 18.5–24.9 kg/m^2^ (World Health Organization, 2019). However, BMI is influenced by age and gender (Nevill and Metsios, 2015) and, in Western societies, is typically higher in men than in women (Kromeyer-Hauschild et al., 2001; Finucane et al., 2011; Statistisches Bundesamt [DESTATIS], 2017). Therefore, in order to examine average-weight women and men who are most representative for their age, we defined our BMI range from 18.5–30 kg/m^2^. We assessed 64 women and 64 men from a student population. Four women and one man were excluded as they did not fulfill the BMI criterion, and three women and nine men were excluded due to insufficient picture quality of their individual stimuli (brightness contrast, size problems). Thus, 57 women and 54 men were included in the analysis. To determine the extent of eating pathology and body image disturbances in the sample, the following questionnaires were answered by all participants.

### Questionnaires

#### Body Appreciation Scale

Body appreciation was assessed using the Body Appreciation Scale (BAS-2; Tylka and Wood-Barcalow, 2015; Behrend and Warschburger, 2022), which consists of 10 items rated on a 5-point scale ranging from never (1) to always (5). The original version yielded a Cronbach’s α of 0.97 for women and 0.96 for men among college and online community samples (Tylka and Wood-Barcalow, 2015). In the present sample, Cronbach’s α was α = 0.88 for women and α = 0.83 for men.

#### Drive for Muscularity Scale

Drive for muscularity was measured using the Drive for Muscularity Scale (DMS; McCreary and Sasse, 2000; Waldorf et al., 2014). The 15 items are rated on a 6-point scale ranging from always (1) to never (6). Items are recoded such that higher scores indicate higher levels of drive for muscularity. Cronbach’s α for the original version of the DMS was 0.82 for women and 0.87 for men in a high school and college student sample (McCreary et al., 2004). The German-language version of the DMS yielded a Cronbach’s α of 0.90 in a non-clinical sample of weight-training men (Waldorf et al., 2014). In the present sample, Cronbach’s α was α = 0.85 for women and α = 0.87 for men.

#### Eating Disorder Inventory-2

Body Dissatisfaction and Drive for Thinness were measured using the two respective subscales of the Eating Disorder Inventory-2 (EDI-2; Garner, 1991; Paul and Thiel, 2005) with seven items for Drive for Thinness and nine items for Body Dissatisfaction. Items of both subscales are rated on a 6-point Likert scale ranging from never (1) to always (6). Analyses of the German-language version of the EDI-2 yielded Cronbach’s α of 0.89 for women and 0.84 for men for the Body Dissatisfaction subscale, and Cronbach’s α of 0.86 for women and 0.70 for men for the Drive for Thinness scale, in a sample of women and men (Paul and Thiel, 2005). In the current sample, Cronbach’s α for women and men were α = 0.89 and α = 0.82, respectively, for Drive for Thinness, and α = 0.89 and α = 0.78, respectively, for Body Dissatisfaction.

#### Eating Disorder Examination-Questionnaire

Eating pathology was assessed using the Eating Disorder Examination-Questionnaire (EDE-Q; Fairburn and Cooper, 1993; Hilbert and Tuschen-Caffier, 2016) which comprises the four subscales, namely, Restraint, Eating Concern, Weight Concern, and Shape Concern. The EDE-Q refers to the last 28 days and contains 22 items which are rated on a 7-point Likert scale from no days/not at all (0) to every day/markedly (6). Cronbach’s α of the German version of the EDE-Q were 0.94 for women and 0.91 for men in a representative German population sample of women and men (Hilbert et al., 2012). In the current sample, Cronbach’s α was α = 0.95 for women and α = 0.88 for men.

### Stimulus Material

Standardized stimulus material was created using photographs of three female and male bodies (ideal, average-weight, overweight) along with one female and one male head. Like the participants, all female and male models providing the face and body stimuli had to be aged between 18 and 30 years. The woman providing the female face was 29 years old and the man providing the male face was 22 years old. As studies suggest a very thin female body and an athletic V-shaped male body as body ideals (Crossley et al., 2012), the ideal bodies were chosen according to these characteristics. For women, the ideal body had a body height of 1.63 m, a body weight of 48.6 kg, a BMI of 18.27 kg/m^2^, and an age of 22 years. For men, the ideal body was 1.88 m tall with a body weight of 91.0 kg, a BMI of 25.78 kg/m^2^, and an age of 25 years. Furthermore, the ideal male body was trained and had a visible six pack. The female and the male average-weight bodies broadly correspond to the average BMI of women and men aged 18–30 years according to the German Federal Statistical Office (Statistisches Bundesamt [DESTATIS], 2017). For women, the average-weight body had a body height of 1.62 m, a body weight of 61.8 kg, a BMI of 23.59 kg/m^2^, and an age of 23 years. For men, the average-weight body had a body height of 1.82 m, a body weight of 80.1 kg, a BMI of 24.20 kg/m^2^, and an age of 20 years, and was normal and untrained. For women, the overweight body had a body height of 1.67 m, a body weight of 72.8 kg, a BMI of 26.09 kg/m^2^, and an age of 25 years. For men, the overweight body had a body height of 1.82 m, a body weight of 95.9 kg, a BMI of 28.97 kg/m^2^, an age of 25 years, and had an untrained stature. Both overweight bodies thus correspond to an overweight body (BMI of 25.0–29.9 kg/m^2^) according to WHO (World Health Organization, 2019). The persons providing the stimulus material gave written consent for the use of their photographs in this study and received 100 Euros for the body pictures and 50 Euros for the face pictures as reimbursement.

**FIGURE 1:**
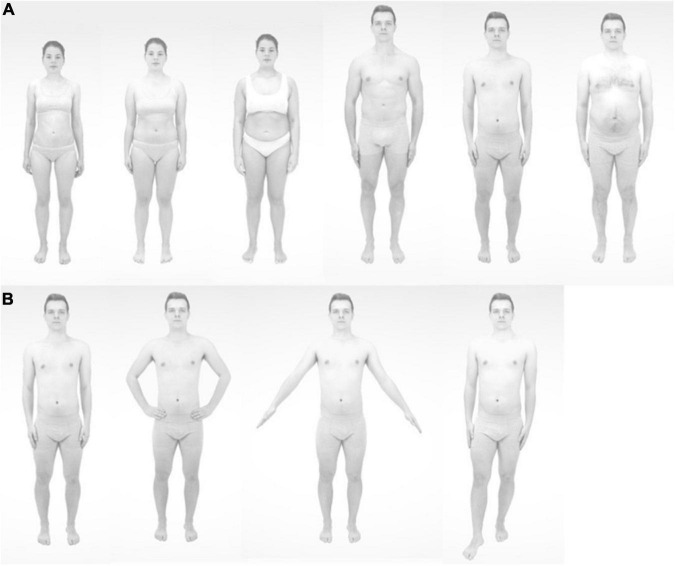
The ideal, average-weight, and overweight female and male bodies **(A)** and the four poses **(B)**.

For the creation of the stimulus material, each individual was photographed in provided gray underwear in a frontal view in front of a white background in four standardized poses with a neutral facial expression (see Figure 1). Using the free software GNU Image Manipulation Program (GIMP), we cut out the bodies and the faces, including hair. With the software MATLAB R2019a (MathWorks; Natick, MA, United States), the female face, the three female bodies, the male face, and the three male bodies were gray-scaled and merged. For each picture, small discrepancies in the throat area were corrected by the functions Clone and Heal in GIMP.

To create the individual stimulus material for each participant, photographs of each participant were taken in the same manner as for the “other” faces and bodies. Similarly, the participant’s face was placed on the same-sex average-weight, overweight, and ideal body stimuli, and the same-sex “other” face was placed on the participant’s body, using GIMP and MATLAB R2019a. In total, for each participant, we constructed 16 body stimuli with the participant’s face (i.e., average-weight, overweight, ideal, participant’s body, each in four poses), 16 same-sex body stimuli with the other face (i.e., average-weight, overweight, ideal, participant’s body, each in four poses), and 12 other-sex body stimuli with the other-sex face (i.e., average-weight, overweight, ideal body, each in four poses). These latter body stimuli from the other gender were shown in order to reduce prompt picture repetition and memory effects. The female and male body stimuli with the other woman’s face, the other man’s face, and the four poses are presented in Figure 1.

### Procedure

First, participants were orally informed about the study. The then gave their written informed consent in accordance with the Declaration of Helsinki. Next, participants put on the provided underwear unobserved in a separate room, and the photos were taken. After the photoshoot, participants’ weight and height were measured. Then, participants changed clothes and were guided to a separate room where they completed the questionnaires through an online survey in Unipark (QuestbackGmbH; Cologne, Germany). At the same time, the investigator edited the participant’s pictures and created the individual stimulus material. Following this, the participants were presented with the body stimuli using the experimental software E-Prime^®^ 3.0 (Psychology Software Tools, Inc.; Sharpsburg, PA, United States). They were told that in some cases, their own faces would be shown on the bodies and that this should help them to identify with the bodies. The body stimuli were presented in randomized order. After a five-second presentation of each 1,920 × 1,080 px body stimulus, participants rated their spontaneous emotional reaction by valence and arousal and rated the body according to body attractiveness, body fat, and muscle mass. All ratings were made on a 9-point Likert scale anchored by the labels very negative—very positive, very calm—very arousing, very unattractive—very attractive, very little body fat—very much body fat, very little muscle mass—very much muscle mass. All participants received student participant credit or monetary compensation (15 Euros).

### Statistical Analysis

Statistical analyses were conducted using SPSS Statistics (IBM; Armonk, NY, United States). To check whether the body stimuli were classified as we expected, we ran a 2 × 4 repeated measures Multivariate Analysis of Variance (MANOVA) with the factors Group (i.e., Men, Women) and Build (i.e., Average weight, Overweight, Ideal, and Self). The dependent variables were the absolute ratings of valence, arousal, body attractiveness, body fat, and muscle mass for the bodies with the other’s face on them. The results from these analyses are provided in the Supplementary Material. To examine our hypotheses regarding the application of double standards, we determined double standards by subtracting the ratings of a body with the other person’s face from the ratings of the same body with the participant’s face for each body build. Thus, a double standard score of approximately zero would suggest no use of double standards. The greater the double standard score deviates from zero, the greater is the extent of the participant’s double standard application. To analyze whether the double standards were significantly different from zero, we used 95% confidence intervals of the double standards. To examine how participants’ gender and the build of the presented bodies affected double standards, we ran a 2 × 4 repeated measures MANOVA with the factors Group (i.e., Men, Women) and Build (i.e., Average weight, Overweight, Ideal, Self), and the double standards of valence, arousal, body attractiveness, body fat, and muscle mass as the dependent variables. For *post-hoc* ANOVAs, we applied the Greenhouse-Geisser correction by default. For *post-hoc t*-tests, the Bonferroni correction was used. As measures of effect size, we report partial eta-squared η_*p*_^2^ for analysis of variance. To test whether double standards are associated with Body Dissatisfaction or Body Appreciation, we calculated Spearman correlations between the Body Dissatisfaction score and the double standards and between the Body Appreciation score and the double standards. It was assumed that *r* = 0.10 indicates a small, *r* = 0.30 a medium, and *r* = 0.50 a large effect.

## Results

### Sample Characteristics

All participants were Caucasian, as the vast majority of the population in the area where the study was conducted is Caucasian. Women (*M* = 21.88, *SD* = 2.67) were slightly younger than men [(*M* = 23.24, *SD* = 3.06), *t*_(109)_ = −2.51, *p* = 0.014], and had a slightly lower BMI [(*M*_*f*_ = 21.78, *SD*_*f*_ = 2.78; *M*_*m*_ = 23.21, *SD*_*m*_ = 2.45), *t*_(109)_ = −2.87, *p* = 0.005]. Furthermore, women scored higher on Body Dissatisfaction (*M*_*bd*_ = 3.15, *SD_*bd*_* = 0.99), Drive for Thinness (*M*_*dt*_ = 2.58, *SD*_*dt*_ = 1.05), and Eating Pathology (*M*_*ep*_ = 1.31, *SD*_*ep*_ = 1.06) than men [(*M*_*bd*_ = 2.35, *SD*_*bd*_ = 0.82; *M*_*dt*_ = 1.73, *SD*_*dt*_ = 0.76; *M*_*ep*_ = 0.83, *SD*_*ep*_ = 0.70), *t*_*bd*(109)_ = 4.64, *p* < 0.001, *t*_*dt*(102.36)_ = 4.93, *p* < 0.001, *t*_*ep*(97.48)_ = 2.83, *p* = 0.006]. In contrast, men (*M* = 2.77, *SD* = 0.84) scored higher on Drive for Muscularity than women [(*M* = 2.13, *SD* = 0.68), *t*_(109)_ = −4.44, *p* < 0.001]. Women (*M* = 3.62, *SD* = 0.57) and men (*M* = 3.77, *SD* = 0.55) did not differ in Body Appreciation [*t*_(109)_ = −1.46, *p* = 0.148]. Furthermore, women and men did not differ in the highest level of educational attainment (lower-track secondary school certificate: 0 women, 1 man; medium-track school-leaving certificate: 0 women, 2 men; advanced technical college entrance qualification: 1 woman, 1 man; higher education entrance qualification: 50 women, 37 men; technical college degree: 1 woman, 3 men; university degree: 5 women, 10 men), χ^2^_(5)_ = 7.53, *p* = 0.184. In sum, the sample consisted of young, highly educated, average-weight, Caucasian women and men with low levels of eating pathology and body image disturbances, very similar to the sample in a previous study on double standards in body evaluation employing cartoon-like bodies as stimuli (Voges et al., 2019b).

### Double Standards

**FIGURE 2:**
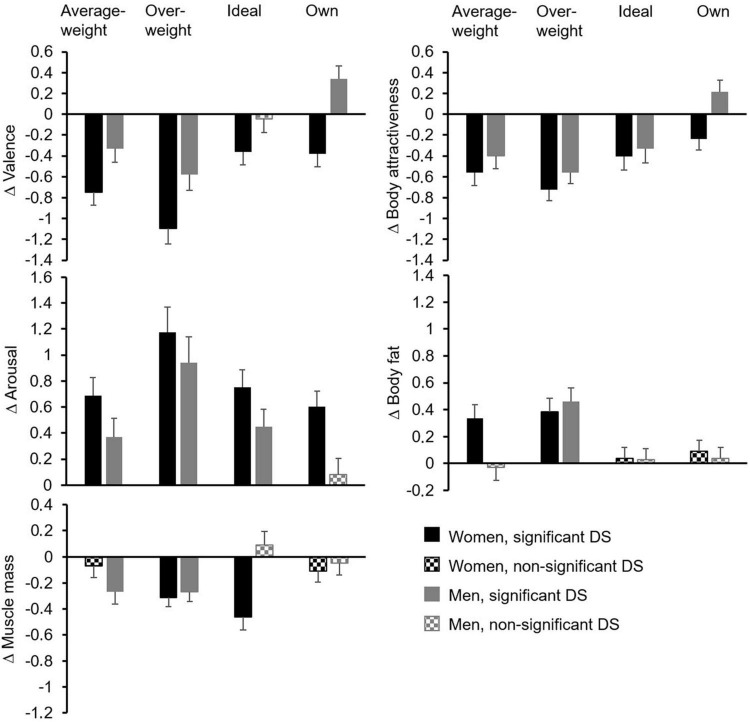
The means and standard errors of the double standards (DS) dependent on Group and Build.

Means and standard errors of the double standards are presented in Table 1 and Figure 2. The MANOVA revealed a main effect of the factor Group [Pillai’s trace = 0.16, *F*_(5,105)_ = 4.04, *p* = 0.002, η_*p*_^2^ = 0.16], a main effect of the factor Build [Pillai’s trace = 0.48, *F*_(15,95)_ = 5.94, *p* < 0.001, η_*p*_^2^ = 0.48], and a significant interaction of Build and Group [Pillai’s trace = 0.35, *F*_(15,95)_ = 3.40, *p* < 0.001, η_*p*_^2^ = 0.35].

**TABLE 1:** Means, standard errors, confidence intervals of the means, and results for the *post-hoc t*-tests for the double standards dependent on the factors Group and Build.

	Women		Men		Over both groups	
			
Variables	*M*	SE	*M*	SE	*M*	SE
**DS valence**						
Average-weight	−0.750*	0.123	−0.333*	0.126	−0.542^*def^	0.088
Overweight	−1.096*	0.146	−0.579*	0.150	−0.838^*cef^	0.105
Ideal	−0.360*	0.126	–0.046	0.130	−0.203^*cd^	0.090
Own	−0.382*	0.124	0.338*	0.127	−0.022^cd^	0.089
Over all builds	−0.647^*b^	0.080	−0.155^a^	0.082	−0.401*	0.057
**DS arousal**						
Average-weight	0.689*	0.139	0.370*	0.142	0.529^*d^	0.099
Overweight	1.175*	0.193	0.940*	0.198	1.058^*cef^	0.138
Ideal	0.754*	0.130	0.449*	0.134	0.602^*d^	0.093
Own	0.601*	0.121	0.079	0.125	0.340^*d^	0.087
Over all builds	0.805^*b^	0.114	0.459^*a^	0.117	0.632*	0.082
**DS body attractiveness**						
Average-weight	−0.561*	0.122	−0.398*	0.126	−0.480^*f^	0.088
Overweight	−0.724*	0.105	−0.560*	0.108	−0.642^*f^	0.075
Ideal	−0.404*	0.132	−0.329*	0.136	−0.366^*f^	0.095
Own	−0.237*	0.105	0.218*	0.108	−0.010^cde^	0.075
Over all builds	−0.481^*b^	0.071	−0.267^*a^	0.073	−0.374*	0.051
**DS body fat**						
Average-weight	0.338*	0.097	–0.028	0.099	0.155^*d^	0.069
Overweight	0.386*	0.098	0.463*	0.101	0.424^*cef^	0.071
Ideal	0.039	0.083	0.028	0.083	0.034^d^	0.058
Own	0.092	0.081	0.037	0.083	0.065^d^	0.058
Over all builds	0.214*	0.051	0.125*	0.052	0.632*	0.082
**DS muscle mass**						
Average-weight	−0.070^e^	0.089	−0.269^*e^	0.091	−0.169*	0.063
Overweight	−0.311*	0.072	−0.269^*e^	0.074	−0.290*	0.051
Ideal	−0.461^*bcf^	0.097	0.093^acd^	0.100	−0.184*	0.070
Own	−0.110^e^	0.083	–0.051	0.085	–0.080	0.059
Over all builds	−0.238*	0.052	−0.124*	0.054	−0.401*	0.057

DS, double standard; M, mean; SE, standard errors that were used for calculation of the 95% confidence interval for each DS. *Zero is out of 95% confidence interval. ^a^Differs significantly from women. ^b^Differs significantly from men. ^c^Differs significantly from the average-weight build. ^d^Differs significantly from the overweight build. ^e^Differs significantly from the ideal build. ^f^Differs significantly from the own build.

*Post-hoc* ANOVAs revealed significant main effects of the factor Group for double standards in valence [*F*_(1,109)_ = 18.44, *p* < 0.001, η_*p*_^2^ = 0.15], double standards in arousal [*F*_(1,109)_ = 4.45, *p* = 0.037, η_*p*_^2^ = 0.04], and double standards in body attractiveness [*F*_(1,109)_ = 4.45, *p* = 0.037, η_*p*_^2^ = 0.04]. Main effects of the factor Group for double standards in body fat [*F*_(1,109)_ = 1.48, *p* = 0.227, η_*p*_^2^ = 0.01] and muscle mass [*F*_(1,109)_ = 2.32, *p* = 0.131, η_*p*_^2^ = 0.02] were not significant. *Post-hoc* ANOVAs also yielded significant main effects of Build for double standards in valence [*F*_(2.84,309.01)_ = 18.15, *p* < 0.001, η_*p*_^2^ = 0.14], double standards in arousal [*F*_(2.65, 288.90)_ = 15.04, *p* < 0.001, η_*p*_^2^ = 0.12], double standards in body attractiveness [*F*_(2.92,318.60)_ = 12.24, *p* < 0.001, η_*p*_^2^ = 0.10], and double standards in body fat [*F*_(2.94,320.18)_ = 8.46, *p* < 0.001, η_*p*_^2^ = 0.07], while no significant main effect of Build for the double standards in muscle mass emerged [*F*_(2.84,309.67)_ = 2.34, *p* = 0.077, η_*p*_^2^ = 0.02]. Furthermore, *post-hoc* ANOVAs yielded a significant interaction effect of Group and Build for double standards in muscle mass [*F*_(2.84,309.67)_ = 7.87, *p* < 0.001, η_*p*_^2^ = 0.07]. The interaction effect of Group and Build for double standards in body fat failed to reach statistical significance [*F*_(2.94,320.18)_ = 2.48, *p* = 0.062, η_*p*_^2^ = 0.02]. No interaction effects of Group and Build for double standards in valence [*F*_(2.84,309.01)_ = 1.04, *p* = 0.373, η_*p*_^2^ = 0.01], arousal [*F*_(2.65,288.90)_ = 0.62, *p* = 0.584, η_*p*_^2^ = 0.01], and body attractiveness [*F*_(2.92,318.60)_ = 1.17, *p* = 0.322, η_*p*_^2^ = 0.01] were found.

*Post-hoc t*-tests results are presented in Table 1, in which it is additionally highlighted which double standards are significantly different from zero. With regard to emotional reactions, women showed significant self-deprecating double standards in valence for all bodies, while men only showed self-deprecating double standards in the case of the average-weight and overweight bodies without showing double standards for the ideal body and a self-enhancing double standard in the case of one’s own body. Across all bodies, women showed more pronounced self-deprecating double standards in valence than men. For both women and men, double standards in valence were most self-deprecating in the case of the overweight body, followed by the average-weight body, which revealed significantly more pronounced self-deprecating double standards than the ideal body and one’s own body. Furthermore, women and men showed significant double standards in arousal for all bodies, with the exception that men did not show a double standard in the case of their own body. Thus, both genders had a higher arousal when viewing the body pictures with their own face compared to with the other face. Across all bodies, women displayed more pronounced double standards in arousal than did men. For both genders, double standards in arousal were more pronounced for the overweight body than for all other bodies. In sum, emotional reactions were mostly self-deprecating for women and men, with the exception of more self-enhancing reactions to one’s own body in men. Women reacted in a more self-deprecating manner than did men across all bodies. In addition, the self-deprecating reaction was strongest in the case of the overweight body for both genders.

Regarding body ratings, women showed significant self-deprecating double standards in body attractiveness for all bodies, whereas, men showed self-deprecating double standards for the average-weight, overweight, and ideal body, but a self-enhancing double standard in the case of their own body. Across all bodies, women showed more pronounced self-deprecating double standards in body attractiveness than men. For both women and men, double standards in body attractiveness were more self-deprecating in the case of the average-weight body, the overweight body, and the ideal body than in the case of one’s own body. With respect to body fat ratings, women showed significant self-deprecating double standards for the average-weight body and the overweight body, whereas men only showed a self-deprecating double standard in the case of the overweight body. For both genders, the double standard for the overweight body was more self-deprecating than the double standards for all other bodies. Considering the marginally significant interaction effect for body fat (*p* = 0.062), there was a trend for a difference between women and men in the double standard for the average-weight body. Finally, women showed significant self-deprecating double standards in muscle mass for the overweight body and the ideal body, while men showed self-deprecating double standards in muscle mass for the overweight body and the average-weight body. The double standard in muscle mass for the ideal body was significantly more self-deprecating in women than in men. Furthermore, for women, the double standard for the ideal body was significantly more self-deprecating than the non-existing “null” double standards for the average-weight body and their own body. For men, the double standards for the average-weight body and the overweight body were significantly more self-deprecating than the non-existing “null” double standard for the ideal body. In sum, in line with emotional reactions, attractiveness ratings were mostly self-deprecating for both genders, with the exception of self-enhancing ratings of one’s own body in men. For both genders, self-deprecating double standards regarding body fat and muscle mass were found for the average-weight and overweight bodies.

### Correlations Between Double Standards and Body Image

For women, correlation analysis between double standards and Body Dissatisfaction revealed significant correlations with small to medium effect sizes, indicating that the more pronounced the Body Dissatisfaction, the more self-deprecating the double standards in arousal (*r*_*s*_ = 0.283, *p* = 0.033), valence (*r*_*s*_ = −0.269, *p* = 0.043), and in body fat for one’s own body (*r*_*s*_ = 0.277, *p* = 0.037), and the more self-deprecating the double standards in body fat for the overweight body (*r*_*s*_ = 0.343, *p* = 0.009). Any other correlations between Body Dissatisfaction and double standards for women were non-significant (all *p* > 0.070). For men, correlation analysis likewise revealed significant correlations with small to medium effect sizes, demonstrating that the more pronounced the Body Dissatisfaction, the more self-deprecating the double standards in arousal (*r*_*s*_ = 0.315, *p* = 0.020), body attractiveness (*r*_*s*_ = −0.334, *p* = 0.013), and in body fat for one’s own body (*r*_*s*_ = 0.281, *p* = 0.040). No further significant correlations between Body Dissatisfaction and double standards were found for men (all *p* > 0.062).

Furthermore, for women, correlation analysis between double standards and Body Appreciation yielded a significant finding with a small effect size, indicating that the higher the Body Appreciation, the less self-deprecating the double standards in valence for one’s own body (*r*_*s*_ = 0.266, *p* = 0.045). Any other correlations between Body Appreciation and double standards in women remained non-significant (all *p* > 0.056). For men, correlation analysis revealed medium effects, showing that the higher the Body Appreciation, the more self-enhancing the double standards in body attractiveness (*r*_*s*_ = 0.405, *p* = 0.002) and in valence for one’s own body (*r*_*s*_ = 0.338, *p* = 0.013). No further significant correlations between Body Appreciation and double standards were found for men (all *p* > 0.062).

## Discussion

The present study was conducted to examine whether women differ from men in the application of double standards in body evaluation. Therefore, we presented the participants’ own bodies and average-weight, overweight, and ideal bodies, once with another face and once with the participant’s face. Women and men were asked to evaluate their emotional reaction regarding valence and arousal and to rate the bodies with regard to body attractiveness, body fat, and muscle mass. Double standard application was measured by the difference between the body ratings generated by the different faces.

First, our hypothesis that women and men would apply self-deprecating double standards in the case of an overweight body was confirmed. For both genders, self-deprecating double standards were observed on all dependent variables. Women and men rated their emotional reaction to an overweight body as more negative and with more arousal. They also rated the overweight body as less attractive, with more body fat, and with less muscle mass when the body had their own face compared to another person’s face. The double standards in valence, arousal, and body fat in the case of the overweight body were more self-deprecating than the double standards in valence, arousal, and body fat for the other body builds. These findings are in line with a previous study that employed cartoon-like body stimuli (Voges et al., 2019b) and might be related to negative stereotypes and stigma associated with overweight and obesity in Western society, e.g., obese people are often stigmatized as “careless,” “disorganized,” and “lazy” (Hu et al., 2018). The activation of self-related schemas through the presentation of one’s own face might result in stricter body evaluations, representing the participants’ rejection of overweight for their own bodies (Voges et al., 2019a).

Contrary to our hypothesis, women also showed self-deprecating double standards for other body builds—average-weight, ideal, and one’s own body—which is not in line with the findings of a previous study using cartoon-like body stimuli, which reported that women applied the same standard to the other images (Voges et al., 2019a). In contrast to the use of cartoon-like bodies in previous studies, participants in the present study evaluated real body stimuli (Voges et al., 2019b). This might have led to a better identification with the body stimuli and might have reinforced body schema activation, resulting in more pronounced double standards in women. Furthermore, as the present study also included women with a BMI of 25–30 kg/m^2^, the women had a higher average BMI than those in the aforementioned study, and they had higher body dissatisfaction and eating pathology than the sample in the previous study (Voges et al., 2019a). However, most women in the present sample (about 87%) had an average weight according to the WHO criteria, and the average BMI of 21.78 was, as in the previous study, in the lower-average weight category. Furthermore, body dissatisfaction and eating pathology were at an average and not notably different level compared to norms for young women (Paul and Thiel, 2005; Mond et al., 2006). Thus, the present results suggest that not only women with eating disorders, but also women without eating disorders, might apply stricter standards for themselves than for others regarding body evaluation, which might foster body dissatisfaction.

The hypothesis that men would show self-enhancing double standards in the case of an ideal body and one’s own body can be partially confirmed by the present findings. With the exception of a self-deprecating double standard in body attractiveness, men showed no double standards for the ideal body, which is not in line with previous findings with artificially created bodies (Voges et al., 2019b). However, in the case of one’s own body, men showed self-enhancing double standards in valence and body attractiveness and no self-deprecating double standards. Although men evaluated the ideal body as more attractive than their own body, identification with this body did not lead to self-enhancing double standards. Thus, young men might have internalized the idea that their own body “fits them well” and does not need to correspond to existing male body ideals in society. This would be in line with findings that men do not believe that the ideal male body is more attainable for themselves than for other men, as women do in the case of the female ideal (Buote et al., 2011). Moreover, this idea would further be consistent with the examined correlations of body appreciation and body dissatisfaction with the self-enhancing double standards in men.

Comparing women’s and men’s body evaluations, women rated in a more self-deprecating manner than did men and, in contrast to men, did not show a self-enhancing double standard for one’s own body. Possibly, female and male stereotypes might contribute to such gender differences in body evaluation (Voges et al., 2019b). According to stereotypes concerning male and female characteristics, men should be “independent,” “strong,” and “outstanding,” while women should be “agreeable” and “friendly” (Guimond et al., 2006). Such stereotypes might simplify self-enhancing evaluation patterns in men while hampering them in women (Meyers-Levy and Loken, 2015). In line with this, men engage more in positive body talk than women (Lin et al., 2021), for whom it seems to be normative to engage in negative fat talk, i.e., degrading the body shape and weight of oneself or others (Tompkins et al., 2009). Thus, women might internalize a devaluation of their own body, while men might be more predisposed to upvalue their own body.

To check whether double standards are associated with body dissatisfaction and body appreciation, we conducted correlation analyses. In line with our hypotheses, the results revealed some associations of body dissatisfaction and body appreciation with double standards in women and men. For women, the higher the body dissatisfaction and the lower the body appreciation, the more self-deprecating was the double standard in valence for one’s own body. Furthermore, the higher the body dissatisfaction, the more self-deprecating were the double standards in arousal and body fat for one’s own body and the double standard in body fat for the overweight body. For men, the higher the body dissatisfaction, the less self-enhancing were the double standards in arousal, body attractiveness, and body fat for one’s own body. Furthermore, as mentioned above, the higher the body appreciation, the more self-enhancing were the double standards in valence and body attractiveness for one’s own body. These findings suggest that double standards related to one’s own body are more directly linked to body dissatisfaction and body appreciation than double standards related to other bodies, as most correlations were found for double standards related to one’s own body and not to the other bodies. This corresponds to the notion that the visual representation of one’s own body is influenced by the attitudes toward one’s own body (Williamson et al., 2004; Maister et al., 2020) and that eating pathology is not linked to a generally distorted body perception or cognition but rather to a cognitive–affective distortion in evaluating one’s own body (Behrens et al., 2021). Thus, in addition to stricter standards for oneself, especially in body fat (Voges et al., 2018), eating pathology might be linked to a negative attitude toward one’s own body. With its idiosyncratic characteristics, one’s own body may not match one’s own standard, but may be viewed as more appropriate for other people.

Furthermore, for women, the association with double standards for the overweight body suggests that a stricter disapproval of overweight and obesity for oneself might also foster body dissatisfaction in average-weight women. The fact that no further associations of body dissatisfaction and body appreciation with double standards related to the other bodies (ideal, average-weight, overweight) were observed might also be partially explained by different cognitive reactions to these bodies leading to the same double standard. For example, self-deprecating double standards might emerge in persons with high body dissatisfaction because they generally devalue themselves compared to others and in persons with low body dissatisfaction, because they dislike imagining having the other body, and prefer their own body. Thus, in contrast to the double standards related to one’s own body, the associations between body dissatisfaction or body appreciation and the double standards related to the other bodies might not be so clear.

The present study is the first to examine double standards in body evaluation with photos of bodies including one’s own body and manipulating identification using different faces. By giving a body a different face, we were able to show that identity influences body evaluation differently in women and men. However, some limitations that might restrict the generalizability of the findings should be mentioned. Although the body stimuli were more realistic than those used in a previous study (Voges et al., 2019b), the stimulus material was standardized and gray-scaled, which likely limited the ecological validity. Furthermore, as we used photos of real persons, the bodies naturally differed somewhat in features other than body build (e.g., body height, skin features, body shape). However, for most body build categories, women and men rated the male and female bodies as equally attractive. As we did not assess persons with eating disorders, muscle dysmorphia, or severe body concerns, our results cannot be transferred to these clinical populations. Furthermore, as our study included a photo shoot in which participants wore their underwear, and participants were required to look at photos of their own body, women and men with high body dissatisfaction might have felt too daunted to participate. Based on previous findings with cartoon-like bodies (Voges et al., 2018) and the detected correlations of double standards with body dissatisfaction in this study, it might be assumed that double standards for one’s own body would be more self-deprecating in the case of participants with eating pathology, which should be examined in future studies. Furthermore, samples with a higher BMI, younger or older persons, or persons from different cultures might show different double standards in body evaluation, as body image has been found to differ across the lifespan (Quittkat et al., 2019), across BMI ranges (Calzo et al., 2012), and across cultures (Swami et al., 2010). Thus, the present results provide information about young, highly educated, and average-weight Caucasian women and men, and should be further examined in other samples.

Future studies could use experimental paradigms to clarify which mechanisms result in double standards and whether such double standards in body evaluation play a causal role in body image disturbances. A possible modification of the study design might be to measure eye movements during body evaluation in order to determine whether different identities result in differences in viewing patterns on the same body. Studies indicate that attentional biases may exist depending on identity, especially in individuals with eating disorders (Bauer et al., 2017) or body dysmorphic disorders (Waldorf et al., 2019). Furthermore, following designs for cognitive bias modification training (Dietel et al., 2020), participants could be trained to internalize double standards (e.g., “You have to work harder than others,” “Only the best is good enough for you,” “You have to be slim”), enabling it to be examined whether this manipulation results in more pronounced double standards in body evaluation and in higher body dissatisfaction. In a next step, cognitive bias modification training (Dietel et al., 2020) or evaluative conditioning paradigms (Glashouwer et al., 2018) could also be used to potentially reduce self-deprecating double standards. Furthermore, preventive strategies that emphasize the diversity and positive aspects of bodies, especially for women (Cohen et al., 2021), or seek to prevent widespread dysfunctional behaviors, such as fat talk (Mills and Fuller-Tyszkiewicz, 2017), or promote positive body talk (Alleva et al., 2021) might be promising. In particular, the newer body neutrality movement on social media which encourages women to attach less importance toward physical appearance might be a helpful approach (Cohen et al., 2021), as findings indicate that the evaluation of neutral characteristics is less biased by the identity of the person being assessed than the evaluation of very desirable or undesirable characteristics (John and Robins, 1993).

In sum, the present study extends previous findings of gender differences in applying double standards to self and other body evaluation. Women, relative to men, are self-depreciating. When their own face is attached to differently shaped bodies, they apply stricter standards of attractiveness, which may account for the prevalence of body image disturbances in women.

## Data Availability Statement

The raw data supporting the conclusions of this article will be made available by the authors, without undue reservation.

## Ethics Statement

The studies involving human participants were reviewed and approved by the Osnabrück University Ethics Committee. The patients/participants provided their written informed consent to participate in this study. Written informed consent was obtained from the individual(s) for the publication of any potentially identifiable images or data included in this article.

## Author Contributions

MV and SV: study design. MV: data collection. MV and HQ: data analysis and manuscript preparation. BS: creation of new software used in the work. All authors: substantive revision of the manuscript and read and approved the final manuscript.

## Conflict of Interest

The authors declare that the research was conducted in the absence of any commercial or financial relationships that could be construed as a potential conflict of interest.

## Publisher’s Note

All claims expressed in this article are solely those of the authors and do not necessarily represent those of their affiliated organizations, or those of the publisher, the editors and the reviewers. Any product that may be evaluated in this article, or claim that may be made by its manufacturer, is not guaranteed or endorsed by the publisher.

## Abbreviations

BAS-2: Body Appreciation Scale-2
BMI: body mass index
DMS: Drive for Muscularity Scale
EDE-Q: Eating Disorder Examination-Questionnaire
EDI-2: Eating Disorder Inventory-2.
