# Supplementary material for: Giving a Body a Different Face—How Men and Women Evaluate Their Own Body vs. That of Others
Source: Front Psychol. 2022 May 2;13:853398. doi: 10.3389/fpsyg.2022.853398 (PMC9108869; doi:10.3389/fpsyg.2022.853398)
Supplement: Supplementary file 1 [file Data_Sheet_1.PDF]

Supplementary material

1 Stimulus material check - Absolute stimuli ratings

To check whether the body stimuli were classified as we expected, we ran a 2 × 4 repeated measures MANOVA with the factors Group (i.e., Men, Women) and Build (i.e., Average weight, Overweight, Ideal, Self). The dependent variables were the absolute ratings of valence, arousal, body attractiveness, body fat, and muscle mass for the bodies with the other's face on them. Means and standard errors of the absolute body evaluations with the other face are presented in Table 1 and Figure 1. The MANOVA revealed a main effect of the factor Group, Pillai's trace = 0.55, $F(5, 105) = 25.57$, $p < .001$, $\eta_p^2 = .55$, a main effect of the factor Build, Pillai's trace = 0.93, $F(15, 95) = 79.75$, $p < .001$, $\eta_p^2 = .93$, and a significant interaction of Build and Group, Pillai's trace = 0.74, $F(15, 95) = 17.69$, $p < .001$, $\eta_p^2 = .74$.

Post-hoc ANOVAs yielded significant main effects of the factor Group for arousal ratings, $F(1, 109) = 7.53$, $p = .007$, $\eta_p^2 = .07$, body fat ratings, $F(1, 109) = 14.26$, $p < .001$, $\eta_p^2 = .12$, and muscle mass ratings, $F(1, 109) = 44.79$, $p < .001$, $\eta_p^2 = .29$. Main effects of the factor Group for ratings of valence, $F(1, 109) = 0.50$, $p = .483$, $\eta_p^2 = .01$, and body attractiveness, $F(1, 109) = 1.01$, $p = .317$, $\eta_p^2 = .01$, were not significant. Post-hoc ANOVAs also yielded significant main effects of Build for valence ratings, $F(2.37, 258.04) = 23.60$, $p < .001$, $\eta_p^2 = .18$, arousal ratings, $F(2.60, 283.52) = 27.28$, $p < .001$, $\eta_p^2 = .20$, body attractiveness ratings, $F(2.51, 273.94) = 127.47$, $p < .001$, $\eta_p^2 = .54$, body fat ratings, $F(2.27, 247.25) = 260.52$, $p < .001$, $\eta_p^2 = .71$, and muscle mass ratings, $F(2.59, 282.19) = 121.50$, $p < .001$, $\eta_p^2 = .53$. Furthermore, post-hoc ANOVAs revealed significant interaction effects of Group and Build for valence ratings, $F(2.37, 258.04) = 3.56$, $p = .023$, $\eta_p^2 = .03$, body attractiveness ratings, $F(2.51, 273.94) = 7.74$, $p < .001$, $\eta_p^2 = .07$, body fat ratings, $F(2.27, 247.25) = 28.46$, $p < .001$, $\eta_p^2 = .21$, and muscle mass ratings, $F(2.59, 282.19) = 32.69$, $p < .001$, $\eta_p^2 = .23$. The interaction effect of the factors Group and Build for ratings of arousal was not significant, $F(2.60, 283.52) = 2.21$, $p = .097$, $\eta_p^2 = .02$.

Post-hoc *t*-tests results are presented in Table 1. With regard to emotional reactions, women and men did not differ in valence ratings for the bodies, except for the overweight body, for which women felt more negative than men. In addition, women felt more negative in the case of the overweight body and their own body than in the case of the average-weight and the ideal body. Men felt most negative regarding the overweight body and felt most positive about the ideal body compared to the other bodies. Furthermore, across all bodies, women showed significantly higher arousal. For both women and men, arousal was highest in the case of one's own body followed by the ideal body, for which arousal was higher than for the average-weight and the overweight body.

With regard to the body ratings, women and men did not differ in attractiveness ratings for the various bodies, except for the overweight body, which men rated as less attractive than did women. Women rated the ideal body as the most attractive, followed by their own body and the average-weight body, and the overweight body as least attractive. Men likewise rated the ideal body as the most attractive, followed by their own body, then the average-weight body, and finally the overweight body. Furthermore, compared to men, women rated the average-weight body and their own body as having more body fat, and the ideal body as having less body fat. Women's body fat ratings were highest for the overweight body, followed by the average-weight

body, their own body, and finally the ideal body. Men’s body fat ratings were highest for the overweight body, followed by the average-weight body, and finally the ideal body and their own body. Furthermore, women rated the overweight, ideal, and own bodies as less muscular than did men. According to the women, muscle mass was highest in the case of the ideal body, followed by the average-weight body and their own body and lastly the overweight body. According to the men, muscle mass was highest in the case of the ideal body, followed by their own body, the average-weight body, and lastly the overweight body (see Table 1).

2 Supplementary Figures and Tables

Supplementary Table 1

Means, standard errors and results for the post-hoc t-tests for each rating variable for each body with the other face dependent on the factors Group and Build

	Women		Men		Over both groups	
Variables	*M*	*SE*	*M*	*SE*	*M*	*SE*
Valence						
Average-weight	5.01[d]	0.13	4.70[de]	0.14	4.85[def]	0.10
Overweight	4.64[bce]	0.16	4.18[acef]	0.16	4.41[ce]	0.11
Ideal	5.34[df]	0.15	5.50[cdf]	0.16	5.42[cd]	0.11
Own	4.67[e]	0.16	4.84[de]	0.17	4.76[d]	0.12
Over all builds	4.91	0.11	4.80	0.11	4.86	0.08
Arousal						
Average-weight	3.67	0.20	3.07	0.20	3.37[f]	0.14
Overweight	3.62	0.19	2.87	0.19	3.24[ef]	0.14
Ideal	3.85	0.19	3.34	0.20	3.60[cdf]	0.14
Own	4.47	0.20	3.53	0.20	4.00[cde]	0.14
Over all builds	3.90[b]	0.18	3.20[a]	0.18	3.55	0.13
Body attractiveness						
Average-weight	4.84[de]	0.15	4.46[def]	0.15	4.65[def]	0.11
Overweight	4.05[bcef]	0.16	3.30[acef]	0.16	3.67[cef]	0.11
Ideal	5.97[cdf]	0.15	6.34[cdf]	0.16	6.15[cdf]	0.11
Own	5.02[de]	0.17	5.16[cde]	0.17	5.09[cde]	0.12
Over all builds	4.97	0.11	4.81	0.11	4.89	0.08
Body fat						
Average-weight	5.73[bdef]	0.13	4.24[ade]	0.13	4.98[def]	0.09
Overweight	6.62[cef]	0.12	6.84[cef]	0.12	6.73[cef]	0.08
Ideal	2.77[bcdf]	0.13	3.34[acd]	0.13	3.05[cdf]	0.09
Own	4.99[bcde]	0.19	3.85[ad]	0.19	4.42[cde]	0.13
Over all builds	5.03[b]	0.09	4.57[a]	0.09	4.78	0.06
Muscle mass						
Average-weight	3.58[de]	0.14	3.82[ef]	0.15	3.70[def]	0.10
Overweight	3.07[bcef]	0.15	3.57[aef]	0.16	3.32[dcf]	0.11
Ideal	4.37[bcd]	0.16	6.99[acdf]	0.16	5.68[cdf]	0.11
Own	3.84[bd]	0.18	4.63[acde]	0.18	4.23[cde]	0.13
Over all builds	3.71[b]	0.11	4.75[a]	0.11	4.23	0.08

Note. *M* = Mean, *SE* = standard errors.
[a] differs significantly from women
[b] differs significantly from men
[c] differs significantly from the average-weight build
[d] differs significantly from the overweight build
[e] differs significantly from the ideal build
[f] differs significantly from the own build

2.1 Supplementary Figures

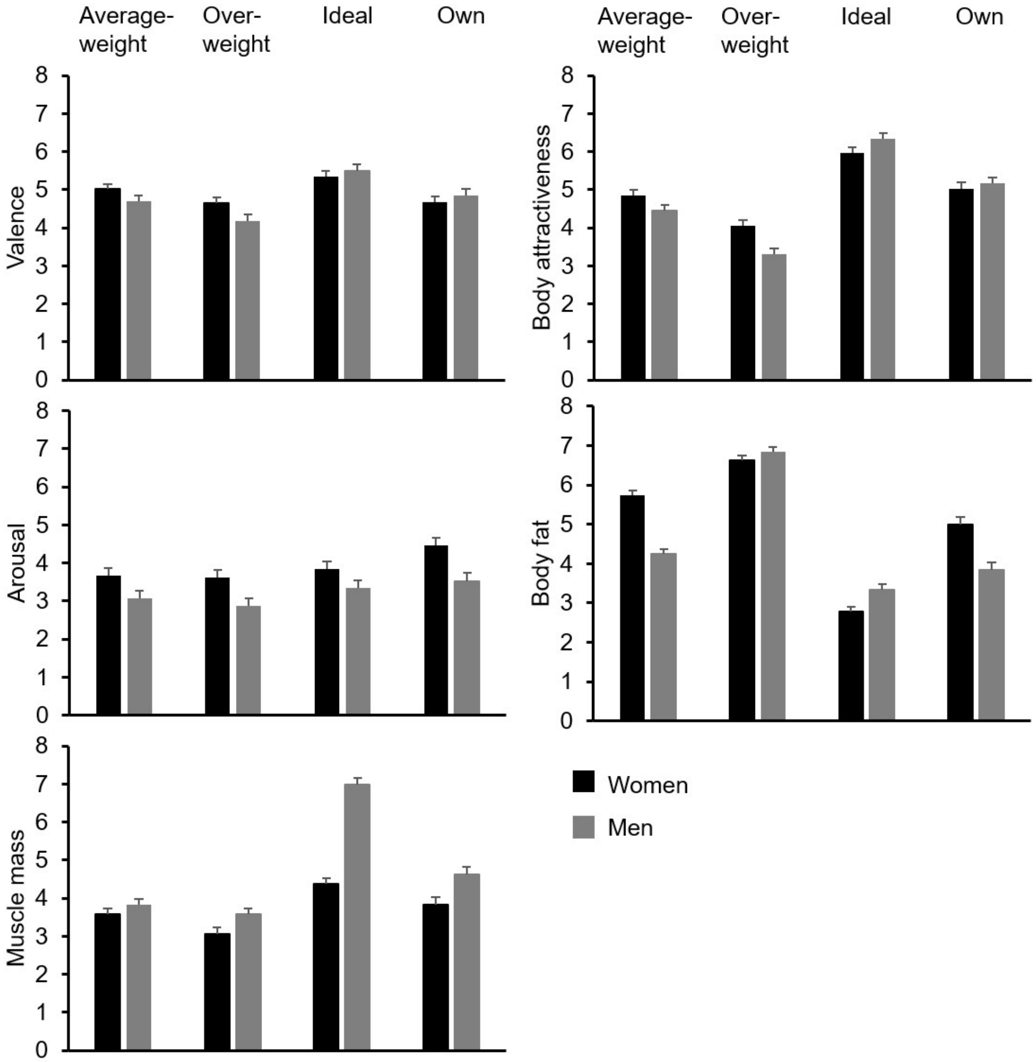

Supplementary Figure 1. The means and standard errors of the ratings for the bodies with the other face
